# COVID-19 Implications on School Dietary Behavior in Chinese College Students: Based on the Longitudinal Assessment of Dietary Records from Intelligent Ordering System

**DOI:** 10.3390/nu17010144

**Published:** 2024-12-31

**Authors:** Shaojie Liu, Hong Peng, Dantong Gu, Mengyun Luo, Haihong Qian, Yingnan Jia

**Affiliations:** 1 School of Public Health, Key Lab of Public Health Safety of the Ministry of Education, Fudan University, 130 Dong’an Road, Shanghai 200032, China; liushaojie@fudan.edu.cn (S.L.); 23211020175@m.fudan.edu.cn (H.P.); 2 Department of Clinical Nutrition, School of Medicine, The First Affiliated Hospital of Xiamen University, Xiamen University, Xiamen 361003, China; 3 Institute of Otolaryngology, Clinical Research Center, Eye and ENT Hospital, Fudan University, Shanghai 200031, China; gdttdg0215@163.com; 4 Prevention Research Collaboration, Sydney School of Public Health, The University of Sydney, Sydney, NSW 2006, Australia; mengyun.luo@sydney.edu.au; 5 Health Communication Institute, Fudan University, Shanghai 200032, China

**Keywords:** coronavirus disease 2019, college students, dietary behavior, intelligent ordering system

## Abstract

Objectives: The coronavirus disease 2019 (COVID-19) pandemic has changed the dietary behavior of college students; however, the persistence of the changes in dietary behavior remains uncertain. This study aims to explore the changes in school food consumption and dietary quality of college students during three distinct COVID-19 periods: pre-epidemic (stage T1), epidemic (stage T2), and post-COVID-19 epidemic (stage T3). Methods: The persistent 6-year data, involving 3,484,081 dietary records from January 2018 to December 2023, for college students were acquired from the “Intelligent Ordering System (IOS)”. School food consumption and total energy intake of each college student per day were evaluated by information on three meals in the IOS combined with the corresponding food database of each dish. The school dietary quality of college students was evaluated by the Chinese Healthy Eating Index (CHEI). Results: In total, 459 college students were included in the T1 period, 530 in the T2 period, and 1316 in the T3 period. At stages T2 and T3, the energy, protein, and fat intakes of college students were higher than those at stage T1 (*p* < 0.001). Meanwhile, the breakfast energy ratio exhibited a significant decrease (0.27 vs. 0.25), while the lunch (0.37 vs. 0.38) and dinner (0.37 vs. 0.38) energy ratios exhibited varying degrees of increase (*p* < 0.001). After the COVID-19 pandemic, the components’ score of the CHEI for dark vegetables, red meats, and sodium showed an increase, while tubers exhibited a decrease (*p* < 0.01). Conclusions: This study provides compelling evidence of the significantly negative impact of the COVID-19 pandemic on school food consumption and dietary quality among Chinese college students. However, the duration of this effect may be limited. There was a certain degree of improvement in the food consumption and school diet quality of college students in China following the conclusion of the epidemic.

## 1. Introduction

Diet plays a crucial role in maintaining human health. An unhealthy diet can augment the incidence of noncommunicable diseases across the globe and has turned into an independent factor that influences the development of metabolic diseases (including obesity, diabetes, hypertension, and dyslipidemia) [1]. Unhealthy dietary habits, including high-fat, high-salt, and high-sugar intakes, can influence the synthesis and accumulation of fats, affect normal glycolipid metabolism, and induce insulin resistance [2,3,4], consequently contributing to the occurrence and development of metabolic diseases [5]. College students are in a critical period to establish a wellness diet habit with the development of individual independence and autonomy [6,7]. Their dietary behaviors established in this period can potentially influence their susceptibility to diet-related illnesses in the future [8]. However, several studies have shown that the dietary habits of college students are still not optimistic, and unhealthy eating behaviors are widespread in college students, such as high intake of fast food, snacks, sweets, and soft drinks, as well as inadequate intake of fruits and vegetables [9,10]. As a transitional life stage from adolescence to young adulthood, college students often face many challenges, including the establishment and development of new dietary behaviors. However, the newly established dietary behaviors of college students are not very stable, which may be influenced easily by individual, social, and environmental factors and sudden life events [11].

The global health emergency status was assigned to the COVID-19 pandemic by the World Health Organization in January 2020 [12]. To prevent the significant surge in the number of confirmed COVID-19 cases, governments implemented diverse strategies to mitigate the spread of COVID-19, such as the wearing of masks and lockdowns to increase social distancing forcefully [13]. A prolonged lockdown strategy may lead to psychological and behavioral alterations for college students [14,15], especially in the forced transformation of dietary patterns due to the lack of a food supply and trouble with food transport [16]. A previous study proved that the diet behavior of college students has been changed strongly by COVID-19, including an increase in soybean, cake, and vegetable intakes and a decrease in fish and grain intakes [7]. With the end of the COVID-19 pandemic and the adjustment of the prevention and control measures of governments, the life of college students is back to normal. However, the persistence of the changes in the dietary behavior of college students induced by COVID-19 remains uncertain. To our best knowledge, no study has evaluated the dietary behavior of college students after the COVID-19 pandemic so as to further evaluate the long-term impact of COVID-19 on the dietary quality of college students.

Currently, the majority of college students have dinner in school canteens. Several studies conducted during the COVID-19 period acquired data on the food consumption of college students by traditional survey methods [7,17], including 3-day, 24 h diet recalls [18], food frequency questionnaire surveys [19], and weighed food records [20]. The aforementioned methods necessitate substantial human, material, and financial resources and fail to adequately capture the long-term trend of dietary change among college students. With the advent of digital technology, lots of novel technologies began to be employed to document dietary data accurately, automatically, and conveniently and resolve several limitations of traditional methods in terms of tracking time and recall bias [21]. In China, this technology has also facilitated the implementation of the “Intelligent Ordering System” (IOS), which encompasses the automatic collection of school eating data, reservation placement, and dish administration [22]. It can provide long-term, consecutive, and relatively accurate meal data for each diner and contribute to identifying the changes in school dietary quality during the COVID-19 pandemic. At present, the school canteens in the Fenglin Campus of Fudan University have witnessed a significant implementation of the IOS since September 2017 [22,23], whose accuracy and effectiveness were verified in a previous study [24]. All meal information of students who dine in the school canteens was consecutively recorded during three distinct periods: pre-epidemic, epidemic, and post-COVID-19 epidemic.

Our research used the persistent 6-year data, involving 3,484,081 dietary records from January 2018 to December 2023, based on the IOS and aimed to (1) explore the changes in the school food consumption, macronutrient intake, and energy supply ratio of three meals during three distinct periods, i.e., pre-epidemic, epidemic, and post-COVID-19 epidemic, for college students in Fudan University’s medical school, China, and (2) further evaluate their changes in school dietary quality by the calculation of the Chinese Healthy Eating Index (CHEI) to identify the impact of COVID-19 on the school dietary quality of college students.

## 2. Materials and Methods

### 2.1. Study Design

**Figure 1:**
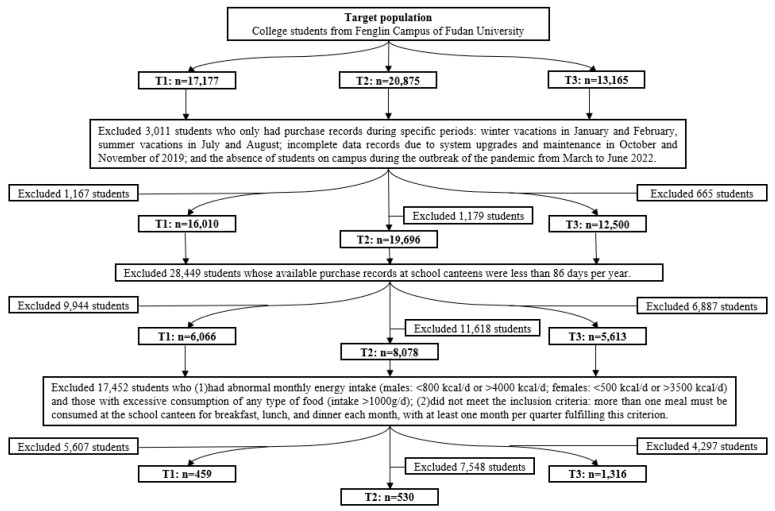
The flow chart of this study.

We adopted a multi-stage cohort study for this study. To assess the changes in school dietary behaviors among college students before and after the COVID-19 pandemic, the enrolled college students were required to have consecutive records to compose three cohorts in the IOS for one of the following three optional COVID-19-related periods: pre-epidemic (T1: January 2018–December 2019), epidemic (T2: January 2020–December 2022), and post-epidemic (T3: January 2023–December 2023). The school food consumption and total energy intake of each college student per day were evaluated by information on three meals in the IOS combined with the corresponding food database of each dish. The average macronutrient intake per month was calculated by the average total intake in effective days in that month. The detailed process was described in our previous studies [22,24]. The periods of January, February, July, and August in each year, corresponding to semester breaks, were excluded from the analysis. Additionally, the period from November 2019 to December 2019 was excluded due to system upgrades in the IOS platform, while the period from March 2020 to June 2020 was excluded since students were not present on campus at the onset of the COVID-19 pandemic. Ultimately, 459 college students were included in the T1 period, 530 in the T2 period, and 1316 in the T3 period. The flow chart of this study is shown in Figure 1. All data in this study were obtained anonymously.

### 2.2. Participants

Targeted participants were enrolled from the Fenglin Campus of Fudan University with medical-related majors, whose meal data were effectively recorded in the IOS. We selected individuals who had a dining attendance of at least 86 days per year in school canteens, with at least one breakfast, one lunch, and one dinner consumed in canteens during each quarter. Individuals with abnormal monthly energy intake (males: <800 kcal/d or >4000 kcal/d; females: <500 kcal/d or >3500 kcal/d) and those with excessive consumption of any type of food (intake > 1000 g/d) were excluded.

### 2.3. Introduction of the IOS

The IOS was implemented at Fudan University’s Fenglin Campus in September 2017 to record students’ dining activities in the canteens. The backend of the IOS inputs detailed common data from dishes, and the merchant displays the corresponding dishes on the system according to the dishes provided by the school canteens per day. The large screen of the system displays a picture of the dish, as well as the name and the price of the dish, for students to view. Once students select their dishes and pay with their student cards, the system automatically generates a dining record. This capability enables continuous long-term monitoring of students’ dining behaviors in the canteens.

### 2.4. Acquiring Dietary Database from the IOS

After the de-identification of personal information, dining records from all consumers eating at the Fenglin Campus of Fudan University from 2018 to 2023 were extracted. These records include names, dining halls, consumption times, consumer type (teachers, students, and support staff), and the types and quantities of selected dishes and associated expenditures for the consumers. We excluded participants other than college students who ate in the canteen, such as teachers. Meanwhile, the research team accurately weighed the amounts of ingredients and condiments used in each dish provided by the dining halls, thereby creating a corresponding food database. The methodology included three key steps [22]: First, before the preparation of each dish, the weights of raw ingredients and condiments were meticulously recorded. Second, the cooked weights of each dish were documented to determine the raw-to-cooked conversion ratios. Third, the cooked dishes in the serving stations of the dining hall were portioned into standardized servings by waiters. Then, five random standardized portions were selected to weigh, with the average values representing the quality of each dish eaten by college students. Utilizing the established food database of each dish, each dining record in the IOS was converted into food groups according to the components of the Chinese Healthy Eating Index (CHEI), such as total grains, fruits, and dairy. Subsequently, macronutrient intakes were calculated using the Chinese Food Composition database (version 2019).

### 2.5. CHEI Calculation

The CHEI is constructed based on the Dietary Guidelines for Chinese (DGC-2016) and serves to differentiate dietary quality among the Chinese population. The total CHEI score is set at 100, comprising 17 components, each contributing a maximum score of either 5 or 10 points [22]. A higher CHEI score indicates greater adherence to the recommended dietary patterns outlined in the DGC-2016, thereby signifying improved dietary quality. Since college students may consume foods like fruits and dairy outside of the canteens, resulting in the misestimation of the intake for such foods, we only calculated the score for 12 specific CHEI components and aggregated the total score (maximum value of 70). According to the CHEI calculation formula, a score achieving at least 60% of the maximum for each component is considered to reach the better level.

### 2.6. Statistical Analysis

The continuous variable age is presented as mean and standard deviation (SD), while the other variables are described as medians (inter-quartile range, IQR). Categorical variables are described using frequencies (ratios). We calculated the percent of food groups complying with the half or general recommendations of the Dietary Guidelines for Chinese Residents among college students. Analysis of Variance (ANOVA) and Chi-square tests were employed to compare demographic characteristics across the different COVID-19 periods. Additionally, Chi-square tests were conducted to evaluate any disparities in the ratios of achieving at least 60% of the maximum for the CHEI score across the three periods. The Kruskal–Wallis test and Mann–Whitney test were utilized to ascertain whether significant differences existed among food group intakes, the energy supply ratios of three meals, macronutrient intakes, and the total CHEI score and its component scores across the three distinct COVID-19 periods, thereby assessing the changes in school food intakes and dietary quality among college students before and after the pandemic. Statistical significance was established by a two-sided *p* < 0.05. All statistical analyses were performed using R version 4.1.1 (Auckland, New Zealand) and Python version 3.7 (Wilmington, DE, USA).

### 2.7. Reporting Guideline Adherence

For this cohort study, we accurately reported the selection criteria for the cohort at baseline, the follow-up procedures over time, and the way we analyzed the changes in the outcome variables, all in line with the stipulations of the STROBE guidelines.

## 3. Results

In total, 459 college students with an average age of 22.8 years were effectively enrolled in stage T1, 530 subjects with an average age of 21.6 years in stage T2, and 1316 subjects with an average age of 23.2 years in stage T3 (overall *p* < 0.001). The proportion of undergraduate students was higher in stages T2 and T3, while the proportion of doctoral students was higher in stage T1 (overall *p* < 0.001). There was no significant difference in the gender distribution among the three distinct COVID-19 periods. The intakes of other food groups, in addition to total grains and soybeans, exhibited significant differences among the three distinct COVID-19 periods (See Table 1).

**Figure 2:**
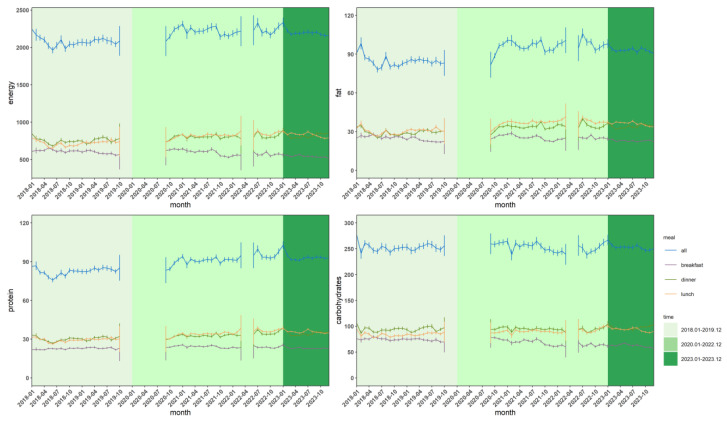
The continuous variation trends in energy and macronutrients for college students from January 2018 to December 2023, stratified by three meals.

The continuous variation trends in energy and macronutrient intakes for college students from January 2018 to December 2023 are shown in Figure 2. Compared to the period before the COVID-19 pandemic, the energy, protein, and fat intakes of college students had an obvious increase during the period of the COVID-19 pandemic. After the end of the pandemic, there was a varying degree of reduction in energy, protein, and fat intakes. There was no obvious change in carbohydrate intake among the three distinct periods. It is worth noting that a certain degree of increase in energy, protein, fat, and carbohydrate intake occurred in January and July per year. Moreover, with the outbreak of the COVID-19 pandemic, the energy, protein, fat, and carbohydrate intakes provided for breakfast exhibited an increasingly significant gap with those provided for lunch and dinner.

Table 2 further compares the differences in the energy, macronutrient, and meal energy supply ratios for college students among the three distinct COVID-19 periods. This result is consistent with Figure 1. After the outbreak of the COVID-19 pandemic (stages T2 and T3), the energy, protein, and fat intakes of college students were higher than those before the pandemic (stage T1), with statistical significance (*p* < 0.001). Meanwhile, the energy supply ratio for breakfast exhibited a significant decrease, while the energy supply ratios for lunch and dinner had varying degrees of increase (*p* < 0.001).

**Figure 3:**
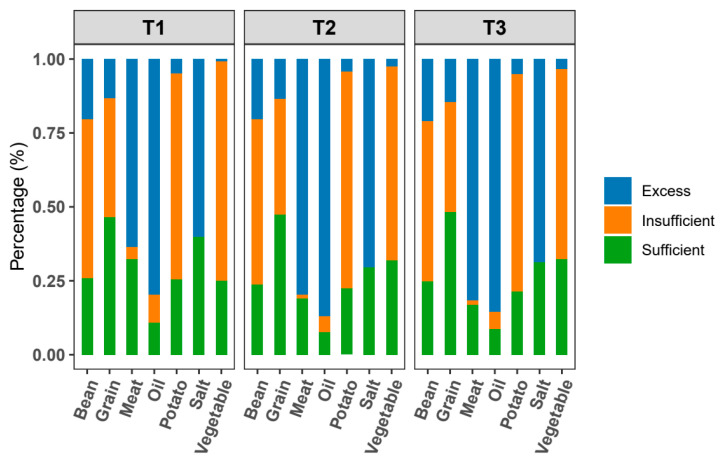
Percentages of college students complying with the half or general recommendation of the Dietary Guidelines for Chinese Residents among the three distinct COVID-19 periods. T1 represents the period before the COVID-19 pandemic (from January 2018 to December 2019); T2 represents the period during the COVID-19 pandemic (from January 2020 to December 2022); T3 represents the period after the COVID-19 pandemic (from January 2023 to December 2023).

Based on the Dietary Guidelines for Chinese Residents, we calculated the percent compliance with the half or general recommendations for college students among the three distinct COVID-19 periods (Figure 3). The percentages of school meat and oil intakes exceeding the recommendations were significantly increased after the outbreak of the COVID-19 pandemic. The percentage of sufficient vegetable intake also increased significantly after the outbreak of the COVID-19 pandemic. However, the proportion of excessive salt intake increased gradually over time among the three distinct COVID-19 periods for college students.

We further evaluated the change in school dietary quality for college students among the three distinct COVID-19 periods by calculating the CHEI, as shown in Table 3. The outbreak of the pandemic did not result in any significant changes in the total CHEI score for college students (*p* = 0.29). However, the comparison before and after the epidemic revealed significant changes in the scores of various components. The scores for dark vegetables, red meats, and sodium showed an increase, while tubers exhibited a decrease (*p* < 0.01). Additionally, there was a fluctuation in the scores of whole grains, mixed beans, and total vegetables, which showed an initial decrease followed by an increase (*p* < 0.01).

Table 4 reveals the ratios for achieving at least 60% of the maximum score for the CHEI and its components in college students among the three distinct COVID-19 periods. The component scores for college students who rarely achieved at least 60% of the maximum included whole grains and mixed beans, total vegetables, fish and seafood, red meats, and cooking oils. Following the pandemic, the increased ratios for achieving at least 60% of the maximum of component scores included whole grains and mixed beans, total vegetables, dark vegetables, fish and seafood, red meats, and cooking oils (*p* < 0.01), while the decreased ratios for achieving at least 60% of the maximum of component scores included total grains, tubers, soybeans, poultry, eggs, and sodium (*p* < 0.01).

## 4. Discussion

In this study, we first adopted a large-scale and long-term cohort study by applying the IOS to describe the variation trend in school food consumption for college students among three distinct COVID-19 periods and then evaluated the changes in school dietary quality for college students undergoing the COVID-19 pandemic through the calculation of the CHEI. This study proved that the COVID-19 pandemic had an obvious implication on school food consumption and dietary quality in Chinese college students.

School food consumption by college students had a significant variation when comparing the pre-COVID-19 epidemic with the COVID-19 epidemic, exhibiting increases in protein and fat for macronutrients and total vegetables, fruits, meat, dairy, salt, cooking oils, and added sugars for food items. The findings of our study partially align with the conclusions drawn in previous studies. Research conducted on university students in Portugal revealed that the lockdown period witnessed increased intakes of fruits and vegetables [25]. Studies conducted in Italy, Latin America, and Romania revealed a similar result to our study during the COVID-19 period. In Italy, 15% of the population turned to farmers or organic sources to purchase fruits and vegetables, particularly in the northern and central regions [26]. A survey involving 6325 participants from five Latin American countries, namely, Brazil, Argentina, Peru, Mexico, and Spain, revealed that 22.7% of the respondents adopted a healthier dietary pattern characterized by increased consumption of fruits, vegetables, and legumes, along with reduced intake of baked products and snacks [27]. Moreover, the pandemic also brought about certain positive impacts on students and employees in Romania, as manifested by the rise in home cooking and the consumption of healthy foods, especially vegetables and fruits [28]. Increasing intakes of protein, fat, and vegetables and fruit rich in vitamins and minerals contribute to maintaining normal immune function, preventing virus infection, and keeping fit [29]. The aforementioned findings suggest that college students demonstrate a commendable level of dietary health literacy when it comes to making informed choices regarding nutritious food for the purpose of maintaining their overall well-being. However, our study showed increased intakes of salt, cooking oils, and added sugars for college students during the COVID-19 pandemic, which was not consistent with a previous study in which Portuguese university students were reported to have adopted healthier eating habits, as evidenced by their self-reported reduction in the consumption of fast food and foods rich in sugar and salt [25]. This may be related to students being able to identify healthy foods but not being able to evaluate the exact amount of oil, salt, and sugar present in foods. Meanwhile, school canteens tend to add more condiments such as oil, salt, and sugar to increase the taste of dishes. The further development of students’ health literacy and a decrease in added condiments in dishes provided by canteens are imperative.

Moreover, the broken line picture showed that college students exhibited a cyclical change in energy, protein, fat, and carbohydrate intakes, with a modest increase in July or January. A possible reason may be that Chinese college students in July and January undergo the final examination at the end of the semester to examine their learning outcomes. At that time, college students face great learning pressure. Several studies have reported that college students with a high level of stress showed an increase in food consumption [30,31], and they may prefer to choose high-calorie, high-fat snack food, sugary food, and carbohydrate-rich food [32,33].

Compared to the school dietary quality before the outbreak of COVID-19, the CHEI score of college students during the COVID-19 pandemic underwent a decreased tendency, encompassing the decreased ratio of achieving at least 60% of the maximum score for the total CHEI score (31.81% vs. 25.28%) and the decreased component scores of whole grains and mixed beans and tubers. Our study exhibited a notable inclination towards the decrease in school dietary quality for college students, aligning with the conclusions drawn from numerous international and regional studies [34,35]. Jehi et al. conducted a systematic review and acquired a similar result, indicating a decrease in the intakes of whole grains and legumes in college students [36]. However, two other studies reported that the COVID-19 period witnessed a shift towards healthier eating habits, including a greater intake of legumes [25,37]. The occurrence of inconsistent results may be attributed to the fact that the foods consumed by college students in this study were provided by the school canteens. The main factor for the decrease in the CHEI score was the decrease in the component scores in whole grains and mixed beans and tubers, which may be related to the diversity decrease in foods provided by the school canteens. Due to the COVID-19 pandemic, the food systems experienced unprecedented disruptions, resulting in shortages of food as well as wastage throughout the supply chain [38]. In China, many regions experienced lockdowns during the pandemic [39], which led to localized food shortages and inadequate supplies. Therefore, school canteens should strengthen food reserves and emergency management. Based on the experience gained during the COVID-19 pandemic, they should increase the reserves of key food ingredients while also taking into account the diversity of reserved foods, ensuring that school canteens can provide a wide variety of food options during special periods.

This study was the first to evaluate the long-term effects of COVID-19 on school food consumption and diet quality of college students after the end of the COVID-19 pandemic. We found that compared to the COVID-19 period, there was a significant improvement in the diet quality of college students after the pandemic, such as the increases in the CHEI component scores in whole grains and mixed beans, total vegetables, dark vegetables, red meats, and sodium. Our study suggests that the school dietary quality of college students had a certain degree of improvement after the pandemic. On the one hand, the school canteens have resumed serving a variety of dishes to ensure food diversity. On the other hand, the subjects of this study were medical college students, who were more aware of nutritional knowledge and health-promoting foods compared to students with other majors [40]. Therefore, when medical students are granted autonomy in food selection, they may exhibit a greater inclination towards choosing health-promoting foods and enhancing food diversity.

Our study also found that the energy supply ratio in three school meals for college students became more unreasonable. The change in the energy supply ratio of three school meals, including the obvious decrease in the energy supply ratio of breakfast over time, is a significant signal of changing school dietary habits for college students, which was not consistent with the Dietary Guidelines for Chinese Residents (the recommendation of the energy supply ratio for three meals is 3:4:3). It suggests that college students are not concerned with their meal allocation throughout the day, especially ignoring the importance of breakfast. Currently, an increasing number of college students are beginning to skip breakfast or eat breakfast casually. In Korea, Kim et al. reported that approximately 56.8% of college students skipped breakfast at least 4 days/week [41]. Similar results also occurred in Saudi medical students, where about 40% of students usually or always skipped breakfast [42]. Breakfast is regarded as the most significant meal of the day, and skipping or neglecting breakfast may increase the risk of multiple diseases, including depression [43], obesity [44], chronic kidney disease [45], and metabolic syndrome [46]. Therefore, emphasis on breakfast consumption and quality in the student population is of utmost importance.

Among the emerging dietary assessment methods, technologies relying on image-assisted approaches require research subjects to take pictures from multiple angles or include standard reference objects in the images so as to enhance the accuracy of dietary intake assessment [47]. Another category of food recording applications developed based on mobile devices can evaluate and record intake according to the standard portion of food. They can also adopt the methods of taking pictures and recording audio, yet still demand relatively high compliance from the subjects [48]. There is also a method that utilizes small wearable cameras to objectively quantify the amount of food, with a measurement error within the range of ±30% [49]. However, none of these methods have been tested on a large scale in the real world. Compared with them, the IOS will not impose an additional recording burden on the subjects. In the future, the IOS can be used to grade dishes based on their health levels and add intuitive nutrition labels to encourage healthier choices. Additionally, it can be widely applied to centralized dining establishments, such as nursing homes, community canteens, and primary and secondary schools, facilitating automated and precise nutritional monitoring.

There are several strengths in this study. The meal data of the school canteen from the IOS were consecutive, relatively objective, and accurate [22]. Our study was the first to apply the persistent 6-year data, involving 3,484,081 dietary records from January 2018 to December 2023, combined with the CHEI scores to relatively precisely evaluate the changes in school food consumption and dietary quality for college students during three distinct periods, i.e., pre-epidemic, epidemic, and post-COVID-19 epidemic, contributing to evaluating the long-term effects of the pandemic. Several limitations are also present in this study. First, the meal data from the IOS cannot provide a comprehensive overview of all the food consumed by individuals in a day. Although a previous study confirmed that meal data from the IOS could roughly reflect individual dietary intake [24], we were unable to capture detailed information on the food intake of students outside of the canteens, such as milk, fruits, and beverages. Second, due to the limitations of the large data in this study, we encountered challenges in gathering more comprehensive information regarding the fundamental characteristics of the study subjects. Consequently, we were unable to conduct further analysis on the potentially influential factors that could impact students’ school dietary quality during the pandemic, such as family economic and social status, health status, physical activity, psychological states, addictive behaviors, and sleeping behaviors [50,51]. Third, the screening principles of college students in this study required them to have complete information on three school meals from the IOS, which may lead to selection bias because of excluding subjects who did not eat a certain meal. However, the screening principles were relatively close to the reality. If the college students who missed a certain meal were included, this may lead to an underestimation of food consumption at school.

## 5. Conclusions

This study provides compelling evidence of the significant and negative impact of the COVID-19 pandemic on school food consumption and dietary quality among Chinese college students. However, the duration of this effect may be limited. In addition to the energy supply ratio in three school meals, the school food consumption and diet quality of college students in China exhibited varying degrees of improvement following the conclusion of the epidemic.

## Figures and Tables

**Table 1:** The demographic characteristics and school food intakes of the study population among the three distinct COVID-19 periods.

Demographic Characteristics		T1	T2	T3	*p* Value
		N = 459	N = 530	N = 1316	
Age	(Mean ± SD)	22.8 ± 2.95	21.6 ± 3.18	23.2 ± 3.12	<0.001 ^abc^
Education levels (n, %)	Undergraduate	155 (33.8%)	295 (55.7%)	684 (52.0%)	<0.001 ^ab^
	Master	103 (22.4%)	76 (14.3%)	228 (17.3%)	
	Doctor	201 (43.8%)	159 (30.0%)	404 (30.7%)	
Gender (n, %)	Male	189 (41.2%)	238 (44.9%)	563 (42.8%)	0.489
	Female	270 (58.8%)	292 (55.1%)	753 (57.2%)	
Tubers (g)	(Median, IQR)	39.6 (28.2, 51.2)	36.0 (23.6, 47.4)	34.6 (22.9, 48.4)	<0.001 ^ab^
Total grains (g)	(Median, IQR)	217 (189, 257)	218 (189, 253)	221 (189, 260)	0.628
Total vegetables (g)	(Median, IQR)	249 (210, 288)	266 (227, 310)	269 (218, 321)	<0.001 ^ab^
Fruits (g)	(Median, IQR)	16.5 (9.38, 26.1)	22.9 (12.7, 35.0)	14.5 (7.06, 25.5)	<0.001 ^abc^
Meat (g)	(Median, IQR)	226 (194, 263)	259 (222, 306)	265 (226, 314)	<0.001 ^ab^
Dairy (mL)	(Median, IQR)	11.7 (4.97, 25.9)	20.0 (9.34, 40.0)	17.3 (7.00, 35.7)	<0.001 ^abc^
Soybeans (g)	(Median, IQR)	24.2 (17.5, 30.7)	23.3 (17.1, 29.9)	24.0 (17.0, 31.8)	0.507
Salt (g)	(Median, IQR)	5.46 (4.76, 6.25)	5.89 (5.14, 6.77)	5.93 (5.00, 6.93)	<0.001 ^ab^
Cooking oils (g)	(Median, IQR)	39.7 (34.0, 45.8)	42.8 (36.7, 48.7)	41.4 (35.8, 48.6)	<0.001 ^abc^
Added sugars (g)	(Median, IQR)	0.22 (0.08, 0.57)	0.33 (0.11, 0.70)	0.32 (0.11, 0.74)	<0.001 ^ab^

T1 represents the period before the COVID-19 pandemic (from January 2018 to December 2019); T2 represents the period during the COVID-19 pandemic (from January 2020 to December 2022); T3 represents the period after the COVID-19 pandemic (from January 2023 to December 2023). *p* ^a^ represents a significant difference comparing T1 with T2; *p* ^b^ represents a significant difference comparing T1 with T3; *p* ^c^ represents a significant difference comparing T2 with T3.

**Table 2:** Comparisons of energy, macronutrients, and meal energy supply ratios for college students among the three distinct COVID-19 periods.

	T1	T2	T3	*p* Value
	N = 459	N = 530	N = 1316	
Energy intake	2030 (1816, 2310)	2152 (1929, 2480)	2141 (1908, 2439)	<0.001 ^ab^
Protein intake	80.1 (72.2, 92.6)	88.8 (78.5, 102)	90.9 (79.9, 104)	<0.001 ^ab^
Fat intake	81.7 (72.4, 94.7)	92.5 (79.9, 109)	90.6 (78.7, 105)	<0.001 ^abc^
Carbohydrate intake	249 (221, 285)	248 (219, 280)	246 (217, 282)	0.195
Breakfast energy ratio	0.29 (0.26, 0.33)	0.27 (0.24, 0.29)	0.25 (0.21, 0.28)	<0.001 ^abc^
Lunch energy ratio	0.35 (0.32, 0.37)	0.37 (0.35, 0.39)	0.38 (0.36, 0.40)	<0.001 ^ab^
Dinner energy ratio	0.36 (0.34, 0.38)	0.36 (0.34, 0.38)	0.37 (0.35, 0.40)	<0.001 ^bc^

Data are shown as the median and inter-quartile range. T1 represents the period before the COVID-19 pandemic (from January 2018 to December 2019); T2 represents the period during the COVID-19 pandemic (from January 2020 to December 2022); T3 represents the period after the COVID-19 pandemic (from January 2023 to December 2023). *p* ^a^ represents a significant difference comparing T1 with T2; *p* ^b^ represents a significant difference comparing T1 with T3; *p* ^c^ represents a significant difference comparing T2 with T3.

**Table 3:** The CHEI and its component scores for college students among the three distinct COVID-19 periods.

CHEI and Component Scores	T1	T2	T3	*p* Values
	N = 459	N = 530	N = 1316	
Whole grains and mixed beans	1.98 (1.67, 2.38)	1.79 (1.48, 2.23)	1.90 (1.47, 2.42)	<0.01 ^abc^
Total grains	5.00 (5.00, 5.00)	5.00 (5.00, 5.00)	5.00 (5.00, 5.00)	<0.01 ^abc^
Tubers	3.34 (2.49, 4.38)	2.63 (1.75, 3.65)	2.06 (1.22, 3.38)	<0.01 ^abc^
Total vegetables	1.40 (1.19, 1.61)	1.38 (1.17, 1.59)	1.46 (1.15, 1.81)	<0.01 ^bc^
Dark vegetables	2.90 (2.33, 3.48)	3.01 (2.41, 3.64)	3.24 (2.46, 4.29)	<0.01 ^bc^
Soybeans	5.00 (5.00, 5.00)	5.00 (4.95, 5.00)	5.00 (4.31, 5.00)	<0.01 ^abc^
Fish and seafood	1.15 (0.67, 1.99)	1.21 (0.70, 1.89)	1.29 (0.52, 2.26)	0.75
Poultry	5.00 (5.00, 5.00)	5.00 (5.00, 5.00)	5.00 (5.00, 5.00)	<0.01 ^abc^
Eggs	5.00 (5.00, 5.00)	5.00 (5.00, 5.00)	5.00 (5.00, 5.00)	0.017 ^c^
Red meats	1.43 (1.15, 1.68)	1.61 (1.34, 1.94)	1.70 (1.27, 2.16)	<0.01 ^abc^
Cooking oils	2.87 (1.66, 4.35)	2.79 (1.54, 4.24)	2.91 (1.45, 5.69)	0.21
Sodium	0.90 (0.35, 10.00)	1.08 (0.50, 10.00)	1.45 (0.65, 10.00)	<0.01 ^bc^
Total scores	39.10 (35.68, 43.17)	38.88 (35.56, 42.10)	38.86 (35.49, 42.89)	0.29

Data are shown as the median and inter-quartile range. T1 represents the period before the COVID-19 pandemic (from January 2018 to December 2019); T2 represents the period during the COVID-19 pandemic (from January 2020 to December 2022); T3 represents the period after the COVID-19 pandemic (from January 2023 to December 2023). *p* ^a^ represents a significant difference comparing T1 with T2; *p* ^b^ represents a significant difference comparing T1 with T3; *p* ^c^ represents a significant difference comparing T2 with T3.

**Table 4:** Ratios for achieving at least 60% of the maximum score for the CHEI and its components in college students among the three distinct COVID-19 periods.

CHEI and Component Scores	T1	T2	T3	*p* Values
	N = 459	N = 530	N = 1316	
Whole grains and mixed beans (≥3, %)	6.97%	3.02%	9.12%	<0.01 ^ac^
Total grains (≥3, %)	100.00%	99.62%	97.57%	<0.01 ^bc^
Tubers (≥3, %)	58.61%	39.81%	30.85%	<0.01 ^abc^
Total vegetables (≥3, %)	0.00%	0.19%	1.98%	<0.01 ^bc^
Dark vegetables (≥3, %)	44.44%	50.75%	58.36%	<0.01 ^bc^
Soybeans (≥3, %)	95.42%	93.58%	87.16%	<0.01 ^bc^
Fish and seafood (≥3, %)	8.28%	6.60%	14.44%	<0.01 ^bc^
Poultry (≥3, %)	99.13%	100.00%	96.05%	<0.01 ^bc^
Eggs (≥3, %)	99.56%	99.81%	98.40%	<0.01 ^c^
Red meats (≥3, %)	0.22%	0.94%	5.09%	<0.01 ^bc^
Cooking oils (≥6, %)	13.73%	13.96%	24.01%	<0.01 ^bc^
Sodium (≥6, %)	35.95%	36.42%	33.28%	0.34
Total scores (≥42, %)	31.81%	25.28%	30.78%	0.036 ^ac^

T1 represents the period before the COVID-19 pandemic (from January 2018 to December 2019); T2 represents the period during the COVID-19 pandemic (from January 2020 to December 2022); T3 represents the period after the COVID-19 pandemic (from January 2023 to December 2023). *p* ^a^ represents a significant difference comparing T1 with T2; *p* ^b^ represents a significant difference comparing T1 with T3; *p* ^c^ represents a significant difference comparing T2 with T3.

## Data Availability

The data presented in this study are available on request from the corresponding author due to privacy.
